# Myoclonus

**DOI:** 10.1002/mdc3.70199

**Published:** 2025-06-27

**Authors:** Masashi Hamada

**Affiliations:** ^1^ Department of Neurology The University of Tokyo Tokyo Japan

**Keywords:** clinical neurophysiology tests, hyperkinetic movement disorders, jerkiness, myoclonus, regularity, tremor

Many digital tools open up new opportunities for a more objective and quantitative assessment of different types of movement disorders. However, not so much has been done in myoclonus. Therefore, this Viewpoint article will focus on classical characteristics and assessment tools, that is clinical neurophysiology testing, for myoclonus, and argue remaining issues regarding diagnosis of myoclonus.

Myoclonus is characterized by brief, shock‐like muscle jerks, caused by abrupt muscle contraction and is produced by abnormal discharges at different levels of the central or peripheral nervous system. Its clinical presentation differs according to its neural generator. The most frequently used classification based on its neural generator comprises four main myoclonus subtypes: cortical, subcortical (including myoclonus associated with dystonia, brainstem myoclonus), spinal (including segmental and propriospinal), and peripheral. This classification heavily relies on the clinical neurophysiology testing, including surface poly‐electromyographic (EMG) recording, long‐latency EMG responses to mixed or cutaneous nerve stimulation (long‐latency reflex [LLR]), electroencephalography (EEG), EEG–EMG polygraphy using back‐averaging (including Bereitschaftspotential [BP]), cortico‐muscular coherence analysis, and somatosensory evoked potentials (SEP). These clinical neurophysiology tests have provided greater clarity to the pathophysiology and the origin of the myoclonus in various diseases.^1^, ^2^, ^3^, ^4^

It is likely that these tests allow us to easily classify the patient's jerky movements into specific types of myoclonus and identify its origin, however, reality is not always that simple in clinical practice. One clinical example of this difficulty is distinguishing myoclonus from tremor.

Tremor and myoclonus are both hyperkinetic movement disorders that typically affect the limbs. First clinical step to differentiate myoclonus and tremor is obviously careful observation, and differentiation is reasonably straightforward^5^; a tremor is defined as an oscillating, rhythmic (ie, regular) movement around a joint,^6^ whereas myoclonus is characterized by brief, shock‐like jerks that appear irregularly.^1^ The key factor for this differentiation is rhythmicity or irregularity. When a patient shows an oscillating, rhythmic and regular movement, it can be easily recognized as a tremor, and when a patient shows irregular brief, shock‐like jerks, we can recognize it as a myoclonus. However, when a patient with tremor shows somewhat irregular components, it can look like a myoclonus, and when a patient shows high frequency myoclonus, it can look like a tremor. In these cases, we are facing the issue regarding the definition of rhythmicity, irregularity, and jerkiness. In other words, tremor is defined by rhythmicity but tremor is not perfectly rhythmic, and myoclonus is defined by jerkiness and irregularity but the definition of jerkiness and irregularity (or regularity) is varied and no consensus has been established.^7^ Furthermore, whether there is a clear border between regularity and irregularity (jerkiness) has not been established.

It is reasonable to investigate these difficult movement disorders using various clinical neurophysiology testing to further characterize the specific features of hyperkinetic movements, but again reality is not always simple. One example is the power spectrum analysis of the movement recording in case of suspected tremor or myoclonus. It is widely recognized that tremor shows a narrow peak in the frequency spectrum analysis and myoclonus shows no clear peak.^5^ However, there are no criteria or consensus for how high and narrow the peak must be.^7^, ^8^ Even the experts may have different opinion when we are looking at the same results of frequency spectrum analysis.^8^, ^9^ Another example is giant SEP in suspected cortical myoclonus patients. Enlarged SEP in myoclonus has been shown to be a clear sign of cortical origin. But the problem is there is no consensus in cut off value and no one knows how large it should be.^2^, ^4^ There is also no consistency on how to measure SEP amplitudes, which could be peak‐to‐peak or baseline to peak nor which components should be enlarged.^2^ Final example in case of myoclonus is that we are sometime facing the situation where not all of clinical neurophysiology tests are supportive to a specific myoclonus type. Even in cortical myoclonus, which might be the most well‐studied type of myoclonus in clinical neurophysiology, it is recently summarized that sensitivity of individual tests may be limited^4^ and no consensus exists which tests or how many tests are necessary to prove or exclude that it is the cortical origin.

What kind of research would be desirable to overcome the issues raised above? For the power spectrogram, for example, it would be ideal to measure it in well‐defined patients with essential tremor (ET) and establish the cutoff value as clinical decision limits to determine how narrow the peak must be. In this case, it is reasonable to use ET definition^8^ for patients’ selection, with further detail examinations (eg, dopaminergic neuroimaging, MIBG scintigraphy, and/or other exams) related to supportive signs or red flags for PD^10^ and MSA^11^ etc. to exclude patients with other than ET. Regarding SEP testing, on the other hand, some authors used to employ the definition of an amplitude greater than the average plus 2 to 3 times the standard deviation (SD) of healthy participants’ responses. This might be better than arbitrary defined giant SEP, but the problem would be it simply reflects upper limit of biological reference values which is different from clinical decision limits.^12^ Thus, establishing cutoff values should be based on the measurements of “giant SEP” in clinically as well as genetically well‐defined patients with cortical myoclonus (such as benign adult familial myoclonic epilepsy, BAFME^13^) without any medication. In both cases, the potential issue would be that these approaches basically premise huge amount of patients’ data which cannot be accomplished by a single institution, and therefore require a framework similar to the World‐Wide Consortium. Finally, to better distinguish tremor from myoclonus, perhaps digital biomarkers capturing fluctuating and rare events using modern technologies might overcome limitations of conventional, snapshot, clinical assessments,^14^ shedding light on a new perspective of tremor or myoclonus, which might possibly contribute to differentiation. Alternatively, given possible pathophysiological mechanisms of BAFME^15^ and progress in this field, a molecular biomarker suggesting cortical origin of myoclonus could be discovered in the near future.

In conclusion, although there are many issues described above, characterizing movement disorders is essential for patients because it directly relates to treatment selection. If we misclassify or misdiagnose patient conditions, patients would lose proper treatment. Thus, clinical neurophysiology testing is necessary to better characterize myoclonus. However, there are quite a few issues in utilizing these tests as well as interpreting the results. As a neurologist, keeping these issues firmly in mind, it would be necessary to deeply think of, sometime conflicting, results of each clinical neurophysiology test to better understand the origin of myoclonus in each patient and to gain some insight into the treatment option. Nevertheless, standardizing the methods and resolving methodological problems as well as establishing diagnostic criteria with high sensitivity and specificity are necessary in future studies.

## Author Roles

(1) Research project: A. Conception, B. Organization, C. Execution; (2) Statistical Analysis: A. Design, B. Execution, C. Review and Critique; (3) Manuscript Preparation: A. Writing of the First Draft, B. Review and Critique.

M.H.: 1A, 1B, 1C, 3A, 3B

## Disclosures

**Ethical Compliance Statement:** The author confirms that, since there were no subjects or patients involved, patient consent or the approval of an institutional review board were not relevant nor required for this work. I confirms that I have read the Journal's position on issues involved in ethical publication and affirm that this work is consistent with those guidelines.

**Funding Sources and Conflict of Interest:** No relevant funding sources or conflicts of interest, given the nature of the article. No off‐label uses of medications or other treatments discussed.

**Financial Disclosures for the Previous 12 Months:** MH received honoraria from the Movement Disorder Society for educational activities and speakers’ honoraria from AbbVie GK, Daiichi Sankyo Co., Ltd., Eisai Co. Ltd., Kyowa Kirin Co. Ltd., Sumitomo Pharma Co. Ltd., Takeda Pharmaceutical Co. Ltd., Ono Pharmaceutical Co. Ltd., and Otsuka Pharmaceutical Co. Ltd.
